# Diagnostic performance of CMR and transthoracic echocardiography in clinical evaluation of cardiac masses with histopathological correlation

**DOI:** 10.1186/1532-429X-15-S1-P59

**Published:** 2013-01-30

**Authors:** Rima D Patel, Ruth P Lim, Muhamed Saric, Ambika Nayar, James Babb, Leon Axel, Iryna Lobach, Monvadi B Srichai

**Affiliations:** 1 Medicine, NYU School of Medicine, New York, NY, USA; 2 Radiology, Austin Health, Melbourne, VIC, Australia

## Background

Echocardiography is the preferred initial imaging method for assessment of cardiac masses. However, CMR, with its excellent tissue characterization and wide field of view, can provide unique information in the evaluation of cardiac masses. Our objective was to identify CMR and echocardiographic parameters that predict the presence of tumor or malignancy in biopsy proven cardiac masses and to assess the potential added value of CMR to echocardiography in the evaluation of cardiac masses.

## Methods

We retrospectively identified 50 patients (50% male, mean age 46 ± 17 years) referred for CMR evaluation of cardiac mass who underwent biopsy or resection with histopathological diagnosis. Echocardiography was performed prior to CMR in 44/50 (88%) of the cases. Echocardiographic and CMR characteristics of the mass were evaluated for their predictive value in distinguishing tumor versus non-tumor and malignant versus non-malignant masses based on histopathology as the gold standard. Tumors were classified as any benign or malignant (primary or metastatic) neoplasm found in or around the heart while non-tumors were classified as any other mass (eg, thrombus) found in this area.Binary logistic regression and ROC curves were used to assess the diagnostic utility of these imaging characteristics alone and in combination for prediction of tumor or malignancy. Wilcoxon rank-sum test was used to compare the number of times a correct pathologic diagnosis was provided by each imaging study.

## Results

The diagnostic performance of echocardiography and CMR parameters found to be significantly predictive of tumor and malignancy on pathology are depicted in the table. A diagnostic model incorporating the aforementioned parameters on echocardiography and CMR found no added value of CMR to echo in the diagnosis of malignant versus non-malignant masses (AUC=.928 vs AUC=.891, p=0.4405). In the 44 cases with both imaging studies, CMR provided significantly more correct pathologic diagnoses compared to echocardiography (77% vs 43%, p<0.0001).

**Table 1:** Diagnostic performance of significant echocardiography and CMR parameters for the detection of malignancy and tumor on pathology

	Accuracy	Sensitivity	Specificity	PPV	NPV
*Echocardiography*					

Tumor (n=31)					

1. Location outside the right atrium	72.7% (32/44)	71.0% (22/31)	76.9% (10/13)	88.0% (22/25)	52.6% (10/19)
2. Location outside the atria and ventricles	50.0% (22/44)	29.0% (9/31)	100% (13/13)	100% (9/9)	37.1%(13/35)

Malignant Tumor (n=17)					

1. Location outside the atria and ventricles	77.3% (34/44)	47.1% (8/17)	96.3% (26/27)	88.9% (8/9)	74.3% (26/35)
2. Non-Mobility	72.7% (32/44)	82.5% (14/17)	66.7% (18/27)	60.9% (14/23)	85.7% (18/21)
3. Pericardial effusion	75.0% (33/44)	52.9% (9/17)	88.9% (24/27)	75.0% (9/12)	75.0% (24/32)

*CMR*					

Tumor (n=35)					

1. Location outside the right atrium	72.0% (36/50)	77.1% (27/35)	60.0% (9/15)	81.8% (27/33)	52.9% (9/17)
2. T2 Hyperintensity or Mixed Pattern	76.0% (38/50)	88.6% (31/35)	46.7% (7/15)	79.5% (31/39)	63.6% (7/11)
3. Contrast Enhancement on First Pass	86.0% (43/50)	82.9% (29/35)	93.3% (14/15)	96.7% (29/30)	70.0% (14/20)
4. Late Gadolinium Enhancement	86.0% (43/50)	85.7% (30/35)	86.7% (13/15)	93.8% (30/32)	72.2% (13/18)

Malignant Tumor (n=21)					

1. Location outside the atria and ventricles	76.0% (38/50)	61.9% (13/21)	86.2% (25/29)	76.5% (13/17)	75.8% (25/33)
2. Non-Mobility	78.0% (39/50)	95.2% (20/21)	65.5% (19/29)	66.7% (20/30)	95.0% (19/20)
3. Contrast Enhancement on First Pass	70.0% (35/50)	85.7% (18/21)	58.6% (17/29)	60.0% (18/30)	85.0% (17/20)
4. Late Gadolinium Enhancement	66.0% (33/50)	85.7% (18/21)	51.7% (15/29)	56.3% (18/32)	83.3% (15/18)
5. Myocardial Invasion	72.0% (36/50)	33.3% (7/21)	100% (29/29)	100% (7/7)	67.4% (29/43)
6. Pericardial Effusion	76.0% (38/50)	47.6% (10/21)	96.6% (28/29)	90.9% (10/11)	71.8% (28/39)

## Conclusions

Although no single or combination of CMR parameters demonstrated significantly improved performance for diagnosing tumor or malignancy over echocardiography, CMR can provide useful information on the underlying histopathologic diagnosis compared to echocardiography alone.

## Funding

NYU School of Medicine

